# Molecular rationale for the use of PI3K/AKT/mTOR pathway inhibitors in combination with crizotinib in *ALK*-mutated neuroblastoma

**DOI:** 10.18632/oncotarget.2372

**Published:** 2014-08-19

**Authors:** Nathan F. Moore, Anna M. Azarova, Namrata Bhatnagar, Kenneth N. Ross, Lauren E. Drake, Stacey Frumm, Qinsong S. Liu, Amanda L. Christie, Takaomi Sanda, Louis Chesler, Andrew L. Kung, Nathanael S. Gray, Kimberly Stegmaier, Rani E. George

**Affiliations:** ^1^ Departments of Pediatric Hematology/Oncology, Dana-Farber Cancer Institute, Boston, MA; ^2^ Cancer Biology, Dana-Farber Cancer Institute, Boston, MA; ^3^ Lurie Family Imaging Center, Dana-Farber Cancer Institute, Boston, MA; ^4^ Broad Institute of MIT and Harvard, Cambridge, MA; ^5^ Departments of Biological Chemistry and Molecular Pharmacology, Harvard Medical School, Boston, MA; ^6^ Institute of Cancer Research, Sutton, United Kingdom; ^7^ Cancer Science Institute of Singapore, Singapore

**Keywords:** ALK, neuroblastoma, crizotinib, mTOR inhibitor, MYCN

## Abstract

Mutations in the *ALK* tyrosine kinase receptor gene represent important therapeutic targets in neuroblastoma, yet their clinical translation has been challenging. The *ALK*^F1174L^ mutation is sensitive to the *ALK* inhibitor crizotinib only at high doses and mediates acquired resistance to crizotinib in *ALK*-translocated cancers. We have shown that the combination of crizotinib and an inhibitor of downstream signaling induces a favorable response in transgenic mice bearing *ALK*^F1174L^*/MYCN*-positive neuroblastoma. Here, we investigated the molecular basis of this effect and assessed whether a similar strategy would be effective in *ALK*-mutated tumors lacking *MYCN* overexpression. We show that in *ALK*-mutated, *MYCN*-amplified neuroblastoma cells, crizotinib alone does not affect mTORC1 activity as indicated by persistent RPS6 phosphorylation. Combined treatment with crizotinib and an ATP-competitive mTOR inhibitor abrogated RPS6 phosphorylation, leading to reduced tumor growth and prolonged survival in *ALK*^F1174L^*/MYCN*-positive models compared to single agent treatment. By contrast, this combination, while inducing mTORC1 downregulation, caused reciprocal upregulation of PI3K activity in *ALK*-mutated cells expressing wild-type *MYCN*. Here, an inhibitor with potency against both mTOR and PI3K was more effective in promoting cytotoxicity when combined with crizotinib. Our findings should enable a more precise selection of molecularly targeted agents for patients with *ALK*-mutated tumors.

## INTRODUCTION

The discovery of activating mutations in the *ALK* gene in high-risk neuroblastoma (NB) has opened new opportunities for the development of novel therapeutic strategies against this often-fatal childhood cancer of the sympathetic nervous system [1]. Tumor cells expressing *ALK* mutations depend on this oncogene for their survival and are typically sensitive to ALK inhibitors such as TAE684 [2, 3]. Crizotinib - the only ALK inhibitor with FDA approval - has limited activity against the various *ALK* mutations identified in patients with NB [4]. Indeed, in a Children's Oncology Group (COG) trial, only 1 of 11 patients with ALK-mutated NB had an objective response to this agent [4]. When tested against NB cells bearing either of the two more common mutations, crizotinib inhibited growth and induced apoptosis in cells expressing *ALK^R1275Q^* but failed to inhibit the growth of *ALK^F1174L^*-positive cells [5, 6]. Similarly, in the COG trial, 3 of the 4 patients with *ALK^F1174L^*–positive NB tumors developed progressive disease, compared with 2 of 5 whose tumors had a missense mutation at the *R^1275^* locus [4]. This relative resistance of *ALK^F1174L^* to crizotinib has been attributed to the increased ATP-binding affinity of the mutant, with complete inhibition of constitutively active ALK attainable only at very high doses of the drug [5]. *ALK^F1174L^* is thus considered the most aggressive of all *ALK* mutations in NB, possessing higher transforming potential and segregating with *MYCN* oncogene amplification, itself a marker of aggressive disease in high-risk NB [7]. Importantly, *ALK^F1174L^* also arises secondarily as a mechanism of resistance after an initial response to crizotinib in patients with *ALK*-rearranged cancers [8].

Although several other compounds are in development or in early-phase trials [9], there is no certainty that these compounds will be any more potent than crizotinib against mutant ALK. Therefore, novel strategies that increase the efficacy of crizotinib in *ALK*-mutated NB, without resorting to doses that could cause irreversible toxicity in children, are particularly relevant. One promising strategy has been the combined use of agents that target not only aberrant ALK, but also other signaling nodes whose activation by mutant ALK contributes to the malignant phenotype [10]. Based on activation of the PI3K/mTOR pathway in a murine transgenic NB model expressing *ALK^F1174L^* and *MYCN*, we demonstrated that combining an ATP-competitive mTOR inhibitor with crizotinib induced tumor regression, although the molecular basis for this result was unclear at the time [6].

The mTOR protein kinase is selectively activated in anaplastic large cell and T-cell lymphomas that are positive for the *NPM-ALK* chromosomal translocation [11]; however, its role in NB cells expressing the full-length mutated ALK receptor remains to be defined. mTOR signaling occurs in the context of at least two multiprotein complexes, mTORC1 and mTORC2, that are key components of the PI3K/AKT network and are activated by growth factors and metabolic status. The mTORC1 complex is a critical mediator of cell growth and metabolism and regulates cell size and protein synthesis through its substrates p70S6K and 4E-BP1 [12]. Activated p70S6K phosphorylates RPS6, an S6 protein of the 40S ribosomal subunit, which in turn causes feedback inhibition of insulin-like growth factor 1 (IGF-1) signaling by phosphorylating insulin receptor substrate 1 (IRS-1), leading to its degradation [13, 14]. The mTORC2 complex, which is also activated by growth factor stimulation, regulates cell proliferation and survival through direct phosphorylation of AKT on serine 473 [12].

Here, we sought to dissect the critical components of *ALK^F1174L^*-associated signaling in the presence of *MYCN* overexpression to identify the molecular determinants of the favorable response to combined crizotinib/mTOR inhibitor therapy previously demonstrated using the TH-ALK^F1174L^/MYCN transgenic model [6]. Moreover, we investigated whether this combination would be just as effective in *ALK-*mutated NB models in the absence of *MYCN* amplification. We show that in cells overexpressing both *ALK^F1174L^* and *MYCN*, there is persistent activation of mTORC1 in the context of single-agent crizotinib treatment. Thus, targeting mTORC1 in combination with ALK leads to enhanced antitumor efficacy and prolongs survival in mouse xenograft models of human NB coexpressing *ALK^F1174L^* and *MYCN*. By contrast, in cells without *MYCN* amplification, this combination, although inducing downregulation of mTORC1, led to reciprocal upregulation of PI3K activity not only in *ALK^F1174L^*-mutated cells but also in those that express the more common *ALK^R1275Q^*-mutation. In this instance, an inhibitor with equal potency against mTOR and PI3K was more effective in promoting cytotoxicity when combined with crizotinib. Our results provide a molecular rationale for the selection of targeted agents to prevent or delay the onset of resistance in patients with *ALK*-mutated NB.

## RESULTS

### PI3K/AKT but not mTOR is inhibited by crizotinib in ALK^F1174L^/*MYCN*-positive NB cells

**Figure 1:**
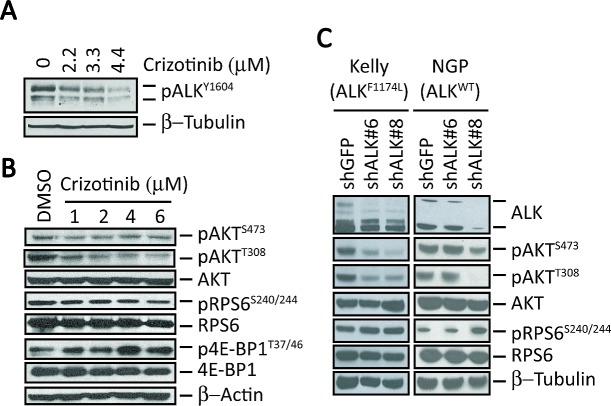
ALK inhibition does not affect mTORC1 signaling in *MYCN*-amplified *ALK^F1174L^*-mutant human NB cells A, Western blot analysis of pALK^Y1604^ in ALK^F1174L^-expressing Kelly NB cells treated with increasing concentrations of crizotinib for 6 hr. B, Western blot analyses of the indicated downstream signaling proteins in Kelly cells after treatment with increasing concentrations of crizotinib for 6 hr. C, Western blot analyses in Kelly (*ALKF1174L*) and NGP (*ALK-wild type*) cells of the indicated signaling proteins in cells in which *ALK* expression was depleted by shRNA knockdown.

To probe the signaling networks in crizotinib-treated NB cells harboring the *ALK^F1174L^* mutation and amplified *MYCN*, we exposed Kelly NB cells (which express both genes) to crizotinib or vehicle for 6 hours and analyzed their gene expression profiles. High doses of crizotinib were required to downregulate phosphorylation of ALK at Y1604 (Fig. 1A). Using comparative marker selection methods and gene set enrichment analysis (GSEA), we identified a number of significantly enriched gene sets in crizotinib-treated cells whose transcripts were functionally linked to protein kinase, insulin receptor, mTOR signaling, MYC, E2F and PTEN pathways. Surprisingly however, the majority of the PI3K/mTOR pathway transcripts were not uniformly downregulated in the crizotinib-treated cells, suggesting inadequate suppression of the pathway by crizotinib even at high doses (Supplementary Table 1).

To determine whether these findings extend to the protein level, we treated Kelly cells with doses of crizotinib similar to those used for the expression analysis and analyzed the three main targets of both mTOR and PI3K signaling: pRPS6 and p4E-BP1, markers of mTORC1 activation, as well as phosphorylation of AKT at serine 473 and threonine 308, markers of mTORC2 and PI3K activation, respectively. We observed that relatively high doses of crizotinib for 6 hours were associated with a decrease in phosphorylation of AKT^T308^ and AKT^S473^ (Fig. 1B). However, pRPS6 was unaffected and p4E-BP1 was even upregulated on exposure to crizotinib (Fig. 1B). Thus, in *MYCN*-amplified NB cells expressing *ALK^F1174L^*, crizotinib effectively downregulated mTORC2 and PI3K, but had no appreciable inhibitory effect on mTORC1 activity.

We next asked whether these differential downstream signaling changes also occur with genetic depletion of mutated ALK. Abrogation of *ALK* expression by stable shRNA transduction led to decreases in pAKT at S473 and T308 but not pRPS6 in Kelly cells. The same phenomenon was observed in *MYCN*-amplified NGP cells that express non-mutated, non-amplified but phosphorylated ALK (Fig. 1C). Together, these studies indicate that in *MYCN*-amplified NB cells, efficient depletion of activated ALK causes differential effects on downstream signaling: PI3K and mTORC2 are downregulated, while mTORC1 activity is maintained.

### *MYCN* amplification determines downstream signaling responses to crizotinib in *ALK*-mutated cells

**Figure 2:**
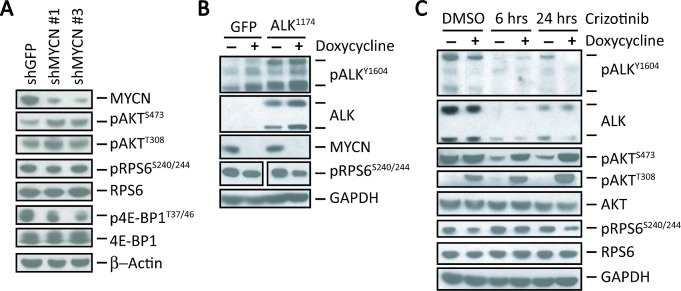
MYCN sustains mTORC1 signaling in NB cells A, Western blot analyses of the indicated signaling proteins in Kelly cells in which MYCN expression was suppressed using shRNA knockdown. B, Composite of western blot analyses of the indicated proteins in SHEP NB cells expressing a doxycycline-repressible *MYCN* construct in which *ALK^F1174L^* was overexpressed using retroviral transduction. Controls are SHEP cells transduced with GFP. Doxycycline (1 μg/ml) was added for 24 hr. to repress MYCN expression. Repression of MYCN led to a 32% reduction in pRPS6 levels compared to cells expressing MYCN (*P =* 0.017) as measured by densitometry. C, Western blot analyses of the indicated proteins in SHEP cells expressing *ALK^F1174L^* with (+) or without (−) *MYCN* repression treated with 1 μM crizotinib for the indicated duration.

The above results suggested that deregulated MYCN could contribute to the sustained upregulation of mTORC1 activity in NB cells expressing activated ALK. To test this hypothesis, we abolished the expression of MYCN in MYCN-amplified, ALK^F1174L^-expressing Kelly cells using shRNA knockdown, and tested whether mTORC1 substrates pRPS6 and p4E-BP1 were affected. Partial knockdown of MYCN that still maintained cell viability did not lead to appreciable changes in pRPS6 levels. However, the phosphorylation of 4E-BP1 at residues associated with mTORC1-mediated inhibition (T37/46) [15] were downregulated at the level of MYCN knockdown achieved (Fig. 2A). Moreover, we observed upregulation of pAKT upon MYCN knockdown (Fig. 2A), consistent with a loss of feedback inhibition associated with reduced mTORC1 activity. To inhibit MYCN expression completely and to further clarify the extent to which it contributes to mTORC1 activity in the presence of mutated ALK, we overexpressed ALK^F1174L^ in the SHEP NB cell line, which stably expresses a tetracycline-repressible MYCN construct (Fig. 2B). Addition of doxycycline to ALK^F1174L^-positive SHEP cells led to depletion of MYCN expression, which was associated with a significant decrease in pRPS6 levels compared with cells in which MYCN was expressed (Fig. 2B). Interestingly, we also noted that pRPS6 expression was decreased in GFP-expressing SHEP cells when MYCN was repressed (Fig. 2B). These data suggest that MYCN contributes to mTORC1 activation.

We next explored the effects of MYCN overexpression in ALK^F1174L^-positive cells upon exposure to crizotinib. As noted previously, we again observed a decrease in pRPS6 levels in DMSO-treated cells when MYCN expression was shut off by the addition of doxycycline (Doxycycline +) (Fig. 2C). Inhibition of MYCN expression in both GFP and ALK^F1174L^-positive SHEP cells led to a decrease in RPS6 phosphorylation compared with results in MYCN-overexpressing cells (p<0.05; Fig. 2C). Crizotinib also led to significant downregulation of pAKT^S473^ levels in MYCN overexpressing cells (Fig. 2C), but not in cells in which MYCN expression was repressed (Doxycycline +), where pAKT^S473^ levels remained elevated. Similarly, MYCN repression led to elevation of pAKT^T308^ (hence PI3K) in SHEP cells, which was further increased on exposure to crizotinib, consistent with continued loss of RPS6-mediated feedback inhibition (Fig. 2C). Thus, in *ALK^F1174L^* cells without *MYCN* overexpression, crizotinib eventually induced downregulation of mTORC1, with both mTORC2 and PI3K remaining activated. In contrast, induction of *MYCN* expression led to downregulation of mTORC2, while sustaining persistent activation of mTORC1. These findings demonstrate that amplified *MYCN* maintains mTORC1 activity in NB cells and contributes to a varied response in AKT activity following crizotinib treatment.

### A selective mTOR inhibitor enhances the effect of crizotinib in *MYCN*-amplified ALK^F1174L^-expressing NB cells

**Figure 3:**
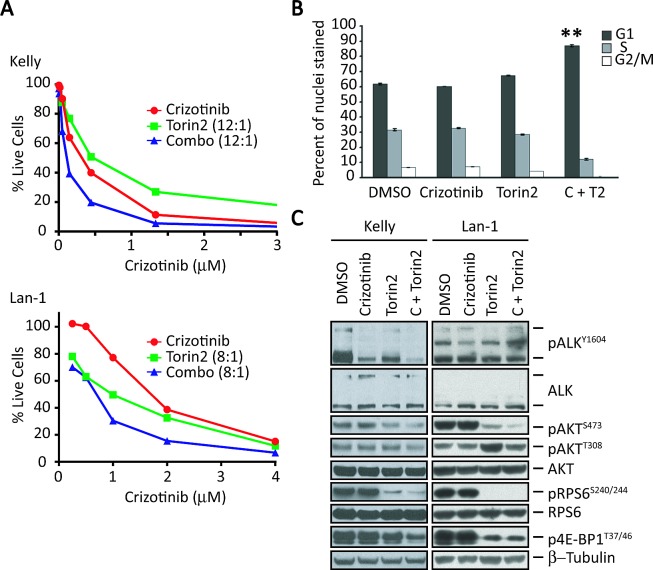
Torin2, a selective mTORC1 inhibitor, enhances the effect of crizotinib in *ALK*^F1174L^-positive, *MYCN*-amplified NB cells A, Survival analysis of *ALK*^F1174L^-positive, *MYCN*-amplified Kelly (upper) and LAN-1 (lower) cells treated for 3 days with increasing concentrations of crizotinib (X-axis), Torin2 (1 nM to 1 μM), or the combination at the indicated ratios (crizotinib:Torin2). B, Cell cycle analysis of crizotinib and Torin2 in Kelly cells. Cells were treated for 72 hr. with vehicle (DMSO), crizotinib (C), Torin2 (T2) or both (C+T2), and analyzed by flow cytometry. The results of three separate experiments are reported as mean ± SEM percentages of cells in each phase of the cell cycle (**P<0.01; Student's unpaired *t*-test). C, Western blot analysis of the indicated proteins in Kelly (left) and LAN-1 (right) cells exposed to crizotinib, Torin2, or the combination. Concentrations of the two agents used together: crizotinib, 1 μM; Torin2, 100 nM.

The above findings suggest that continued or compensatory upregulation of key signaling molecules during crizotinib treatment could contribute to the limited activity of this agent against NB cells expressing *ALK^F1174L^* with concomitant overexpression of *MYCN*. To address this issue, we screened several mTOR, PI3K and dual PI3K/mTOR pathway inhibitors in NB cells (Table S2), identifying Torin1 [16] and Torin2 [17] as promising candidates. Although Torin1, an ATP-competitive mTOR inhibitor [16] with preferential activity against mTOR (EC_50_, 3 nM) compared to PI3K (EC_50_, 1.8 μM), exhibited impressive cytotoxicity with downregulation of mTORC1 and mTORC2 targets in Kelly NB cells (Supplementary Fig. S1A and B), it has a low-yielding synthetic route, poor water solubility, limited oral bioavailability and a short half-life. These restrictions led us to test a second-generation analogue, Torin2 [17], which proved more potent than Torin1 against mTOR (EC_50_, 0.25 nM) as well as PI3K (EC_50_, 200 nM), and led to a decrease in both pAKT^S473^ and pRPS6 levels (Supplementary Fig. S1C). Because of its reported lack of sustained cytotoxic activity as a single agent [17], we next asked whether Torin2 would enhance the sensitivity of *ALK^F1174L^*-expressing NB cells to crizotinib. Indeed, the combination of Torin2 and crizotinib reduced the viability of both Kelly and LAN-1 (*ALK^F1174L^*-positive/*MYCN* amplified) cells beyond that achieved by either single agent alone (Fig. 3A). Growth inhibition in Kelly cells was accompanied by a G_0_-G_1_ cell cycle arrest (Fig. 3B), although a significant increase in apoptosis was not observed (Supplementary Fig. S2A and S2B).

Further testing of this combination in both Kelly and LAN-1 cell lines using synergistic doses of each compound elicited a striking and sustained decrease in phosphorylated RPS6 and 4E-BP1 as well as AKT^S473^, confirming the targeted inhibition of the mTORC1 and mTORC2 complexes (Fig. 3C). Torin2 as a single agent caused an increase in the marker of PI3K activation, pAKT^T308^, suggesting loss of feedback inhibition by mTORC1. However, the combination of Torin2 and crizotinib led to downregulation of pAKT^T308^ in both cell lines (Fig. 3C). Together, these results show that an mTOR-specific inhibitor can augment the cytotoxic activity of crizotinib in *ALK^F1174L^*-mutated NB cells with *MYCN* amplification.

### The combination of crizotinib and Torin2 shows enhanced antitumor activity and prolongs survival in a xenograft model of human *ALK*^F1174L^/*MYCN* –amplified NB

**Figure 4:**
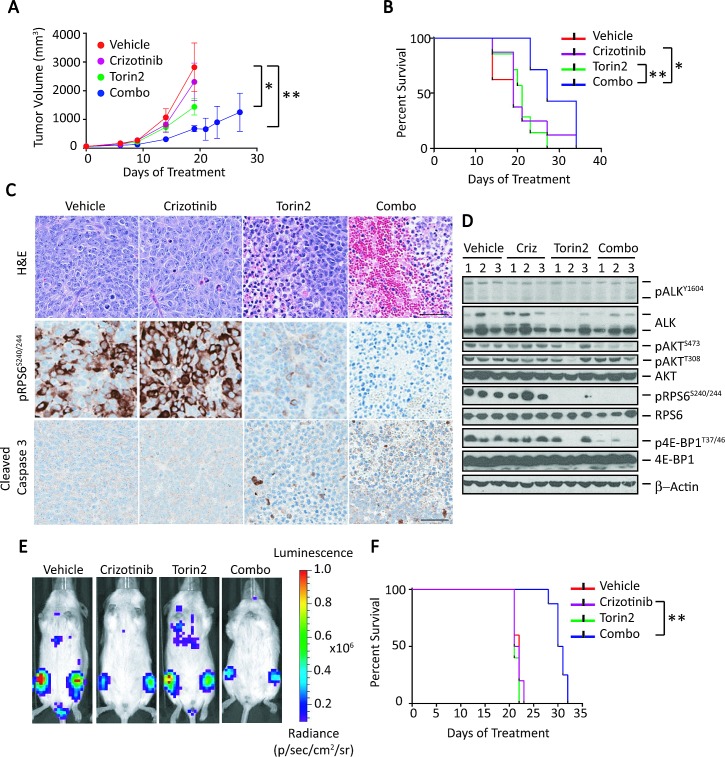
Combination of crizotinib and Torin2 leads to tumor regression and extends survival in an *ALK^F1174L/MYCN^* overexpressing human NB cell xenograft model with attendant effects on downstream signaling A, The growth of Kelly cell tumor xenografts was monitored in mice treated with vehicle, crizotinib, Torin2 or the combination, orally once daily for 3 cycles. Tumor volume measurements were truncated at the time point at which the first animal in a cohort had a tumor volume measuring >1000mm^3^. Tumor volumes are expressed as mean ± SEM. Volume comparisons were performed at day 18 (vehicle vs. crizotinib, P=not significant; vehicle vs. Torin2, *P<0.01; vehicle vs. combination, **P< 0.001; all comparisons by two-way ANOVA). B, Kaplan-Meier survival analysis of mice treated with control, single agents alone or the combination of crizotinib and Torin2 (crizotinib vs. combination, *P=0.03; Torin2 vs. combination, **P=0.005; all by log-rank test). C, Hematoxylin and eosin (H&E), pRPS6 and cleaved caspase-3 immunohistochemical staining on tumor sections following treatment with either single agents or the combination. The scale bar represents 50 μm. D, Immunoblot analysis of the indicated proteins in tumors of mice treated as indicated above. E, Bioluminescence measurements of human NB xenograft models established by intravenous injection of *ALK^F1174L^*/*MYCN*-positive NB cells. Animals were treated with the indicated agents. F, Kaplan-Meier survival analysis of mouse xenograft models of human NB treated with the indicated agents (crizotinib vs. combination, **P=0.001; by log rank test).

Following the positive results obtained from the combination of crizotinib and Torin2 treatment in the transgenic NB model [6], we sought to determine whether similar results would be observed in models of human disease. Therefore, we tested the crizotinib/Torin2 combination in human NB xenograft models generated by subcutaneous injection of Kelly cells into NSG mice. When tumors reached an optimal size (tumor volume, ~60mm^3^), the mice were separated into four groups (n = 8 each) and treated orally once daily with vehicle, crizotinib (75 mg/kg), Torin2 (20 mg/kg), or a combination of the two. Mice were treated in “cycles”, each consisting of 5 days on and 5 days off treatment for a total of three cycles, to mimic clinical use of these drugs as closely as possible. Crizotinib by itself lacked any apparent activity, while Torin2 significantly suppressed tumor growth at 18 days (Fig. 4A), although this result did not translate to prolonged survival (Fig. 4B). Combination treatment, by contrast, significantly attenuated tumor growth and decreased tumor volumes, with minimal toxicity and prolonged survival compared to control animals and cohorts treated with either Torin2 or crizotinib alone (Fig. 4B).

To account for the enhanced activity of crizotinib plus Torin2 in terms of changes in specific signaling molecules, we analyzed post-treatment samples from the subcutaneous xenograft models of *ALK^F1174L^*-mutated NB (n = 3 specimens per group, collected when tumors reached the volume threshold: range, 19-27 days; mean 21 days). Morphological analysis of tumors treated with the combination showed massive hemorrhage and necrosis, in contrast to the negligible or only minor changes in cellular architecture seen with use of either agent alone (Fig. 4C). This paucity of morphologic alterations correlated with a lack of effect of crizotinib on pALK and on the expression of either mTORC1 (pRPS6 and p4E-BP1) or mTORC2 (pAKT^S473^) targets (Fig. 4D). In fact, on immunohistochemical (IHC) staining, pRPS6 appeared to be upregulated in the crizotinib-treated tumors (Fig. 4C). The profile of molecular events seen with single-agent therapy contrasted sharply with that induced by the crizotinib/Torin2 combination. Indeed, there was a striking decrease in AKT^S473^ phosphorylation as well as the virtual disappearance of pRPS6 and p4E-BP1 on western analysis of treated tumor cells (Fig. 4D). This dramatic decrease in mTORC1 signaling was confirmed by IHC showing downregulation of pRPS6 (Fig. 4C). Moreover, the combination also led to an increase in tumor cell death by H&E and cleaved caspase-3 staining, compared with either agent alone (Fig. 4C). Together, these results show that the combination of crizotinib and an mTOR inhibitor can be used both safely and effectively in human NB models.

### Inhibition of ALK and mTOR signaling leads to attenuation of tumor growth and extends survival in a metastatic model of *ALK*^F1174L^/*MYCN* –amplified NB

Given the potency of crizotinib plus Torin2 against subcutaneous xenografted NB tumors, we asked if the combination would be effective in models of metastatic *ALK^F1174L^*/*MYCN*-positive NB. Six-week-old NSG mice were injected with luciferase-expressing Kelly cells by lateral tail vein injection, with bioluminescence used to assess engraftment in the liver 6 days later. At 10 days post injection, repeat imaging revealed increased tumor burden with metastases to many organs, including bone marrow and lungs, reminiscent of human stage 4 NB. At this point, mice were divided into four treatment groups based on similar mean bioluminescence intensities (n = 8 per group) and received vehicle, crizotinib (100 mg/kg), Torin2 (20 mg/kg), or a combination of these agents, given in daily oral doses for 10 consecutive days. The standard dose of crizotinib (100 mg/kg) allowed us to determine its tolerability in combination with other agents. Mice treated with the combination of crizotinib and Torin2 showed signs of toxicity after 6 days of treatment, but these effects resolved rapidly after cessation of treatment for 6 days, at which point the combination was restarted and continued in 5-day on/off cycles as described for the s.c. models. Serial monitoring of tumors by bioluminescence did not reveal any significant differences among the treatment groups until day 21, at which time the cohort treated with crizotinib plus Torin2 had lower bioluminescence compared to mice treated with single agents or vehicle (Fig. 4E and Supplementary Fig. S2C). As in the s.c. models, monotherapy with crizotinib or Torin2 did not appreciably affect the tumor burden or survival in our metastatic model, in contrast to combination therapy, which resulted in significant suppression of tumor growth and prolongation of survival (Fig. 4F). These *in vivo* data support the use of crizotinib with an mTOR catalytic inhibitor in patients with *ALK^F1174L^*-mutated, *MYCN*-amplified tumors.

### The combination of crizotinib and a dual PI3K/mTOR inhibitor is required to induce cytotoxicity in *ALK*^F1174L^-positive NB cells without *MYCN* amplification

**Figure 5:**
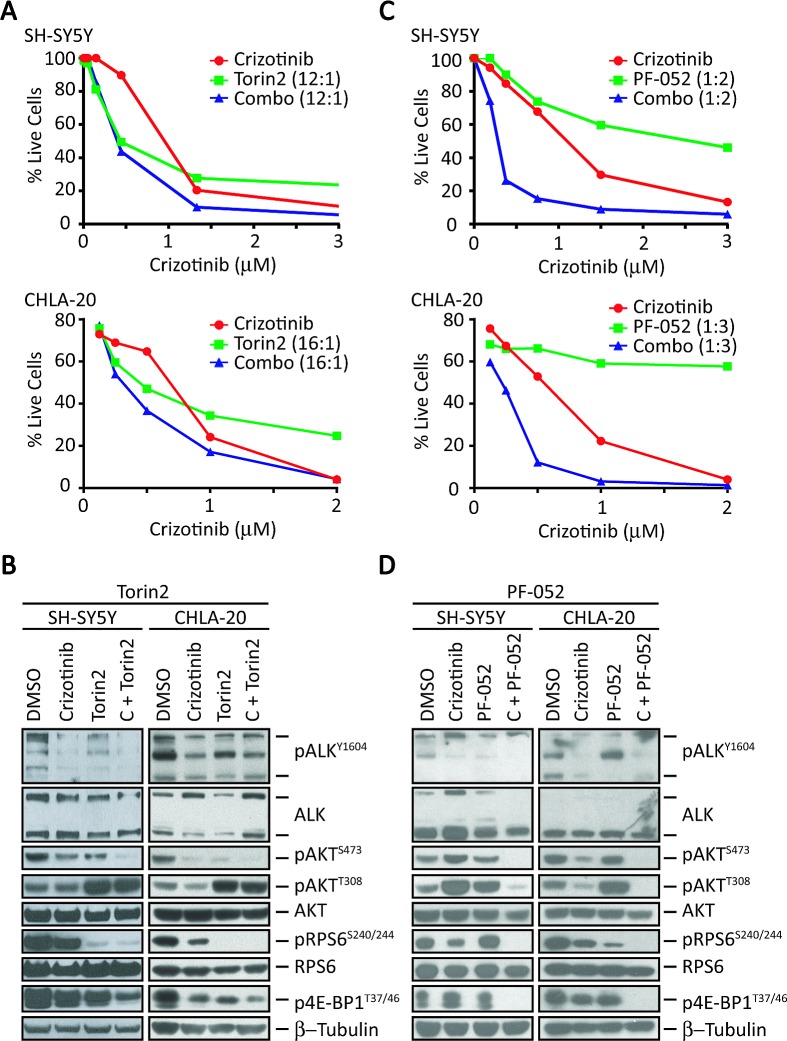
Combination of crizotinib and a dual PI3K/mTOR inhibitor results in synergistic activity in *MYCN*-non-amplified cells A, Survival analysis of SH-SY5Y (top) and CHLA-20 (bottom) cells treated for 3 days with increasing concentrations of crizotinib (x-axis), Torin2 (1 nM to 1 μM), or the combination at the indicated ratios (crizotinib:Torin2). B, Western blot analysis of the indicated proteins in SH-SY5Y (left) and CHLA-20 (right) cells treated at synergistic doses with either vehicle (DMSO), crizotinib (1-3 μM), Torin2 (100 nM), or the combination of crizotinib with the indicated inhibitor for 24 hr. C, Cell survival analysis of SH-SY5Y (top) and CHLA-20 (bottom) cells treated with increasing concentrations of crizotinib (x-axis), PF-05212384 (PF-052, 0.1 nM to 6 μM), or the combination at the indicated ratios (crizotinib:PF-05212384) for 3 days. D, Western blot analysis of the indicated proteins in SH-SY5Y (left) and CHLA-20 (right) cells treated with either vehicle (DMSO), crizotinib (1 μM), PF-05212384 (5 μM) or the combination of crizotinib with the indicated inhibitor for 24 hr.

We next determined whether the combination of crizotinib and Torin2 would be as effective in *ALK^F1174L^*-expressing SH-SY5Y without *MYCN* amplification (Fig. 5A and 5B). In contrast to the single agent crizotinib response in *MYCN* amplified cells (Fig. 1 and Fig. 3C), cells with absent *MYCN* amplification demonstrated reduced pRPS6 and p4E-BP1 expression (Fig. 5B and 5D), which is consistent with the hypothesis that MYCN activity contributes to mTORC1 activation. In these cells, the combination of Torin2 and crizotinib led to a decrease in mTORC1 and mTORC2 signaling as evidenced by downregulated pRPS6, p4E-BP1 and pAKT^473^, respectively (Fig. 5B). However, this was not reflected in synergistic or additive cytotoxicity (Fig. 5A), most likely due to loss of feedback inhibition and the marked reactivation of pAKT^T308^ (Fig. 5B). These data suggested the need for an inhibitor that was also active against PI3K. Hence, we tested PF-05212384, which is extremely potent against both PI3K and mTOR (IC_50_ of 0.4 and 1.6 nM, respectively). When combined with crizotinib, this compound showed synergistic activity in these cells (Fig. 5C and 5D), leading to the downregulation of not only mTORC1 and mTORC2, but also PI3K (Fig. 5D). We further validated these findings in CHLA-20 cells that express the more common R1275Q mutation, suggesting that various ALK mutations respond similarly to the combination in the setting of non-amplified *MYCN*. These findings suggest that mutant *ALK*-expressing NB cells with non-amplified *MYCN* may respond better to an inhibitor that is equally potent against mTOR and PI3K when combined with crizotinib.

## DISCUSSION

The *ALK^F1174L^* mutation, which occurs in NB and cosegregates with the *MYCN* oncogene, demonstrates limited susceptibility to crizotinib both in animal models and in clinical testing [4, 6]. We have previously shown that the combination of crizotinib with an mTOR inhibitor induces responses in transgenic mouse models of NB positive for *ALK^F1174L^* and amplified *MYCN* [6]. Here, we provide a molecular basis for this effect, demonstrating that incomplete inhibition of mTORC1 limits the activity of crizotinib in NB cells that express both *ALK^F1174L^* and deregulated *MYCN*. This interpretation is supported by our observation that concomitant inhibition of mTOR restores sensitivity to crizotinib in NB models. We also show that this combination is not as effective in *ALK*-mutated cells expressing non-amplified *MYCN*. In such cells, an agent with appreciable activity against PI3K as well as mTOR was most effective when combined with crizotinib.

In cells expressing *ALK^F1174L^* and amplified *MYCN*, crizotinib treatment had minimal to no effect on pRPS6 activity, suggesting that MYCN was capable of maintaining mTORC1 activity in this context. This prediction was confirmed in *MYCN*-repressible SHEP NB cells, where overexpression of *MYCN* in the presence of constitutively activated *ALK^F1174L^* led to sustained activation of pRPS6. Moreover, repression or shRNA knockdown of MYCN led to diminished activation of mTORC1 targets. In cells expressing mutant *ALK* and wild-type *MYCN*, moreover, exposure to crizotinib resulted in downregulation of pRPS6 activity, in contrast to *MYCN*-amplified cells where pRPS6 was unaffected, again emphasizing the contribution of deregulated *MYCN* to the sustained activation of mTORC1. Thus, persistence of mTORC1 in the presence of amplified *MYCN* may be a general phenomenon in *ALK^F1174L^*-mutated, *MYCN*-amplified NB tumors, especially since this mutation segregates with *MYCN* amplification [7]. Moreover, it is likely that in these cells, even newer ALK inhibitors with higher potency against the mutant kinase, will be limited by persistent activity of deregulated *MYCN*. We also observed that in cells in which MYCN expression had been abrogated, pAKT^T308^ was upregulated. This reciprocal relationship illustrates the relief of feedback inhibition by mTORC1 and appears to occur not only in NB but in other cancers as well [18, 19]. Such loss of feedback inhibition has also been shown to activate upstream RTKs [13] thus questioning the use of downstream signaling inhibitors as single agents to secure a sustained response.

The ability of MYCN to sustain mTORC1 activity is consistent with previous reports showing that *MYC* overexpression or deregulation activates mTOR signaling [20]. Indeed, *MYC* amplification has been observed more often in cells resistant to PI3K/mTOR inhibitors, as illustrated by the elevated expression of *MYC* and *eIF4E* in cells that have acquired resistance to the mTOR inhibitor BEZ235 [21]. The exact mechanism by which MYCN activates mTORC1 is unclear, but may entail the repression of AMP-activated protein kinase (AMPK) signaling [22] or transcriptional regulation of the negative mTORC1 regulator TSC2 [23]. As previously shown, tumors with high levels of MYCN respond quite dramatically to mTORC1 inhibition, although this property alone does not appear to be sufficient in tumors expressing *ALK^F1174L^* as well [6].

**Figure 6:**
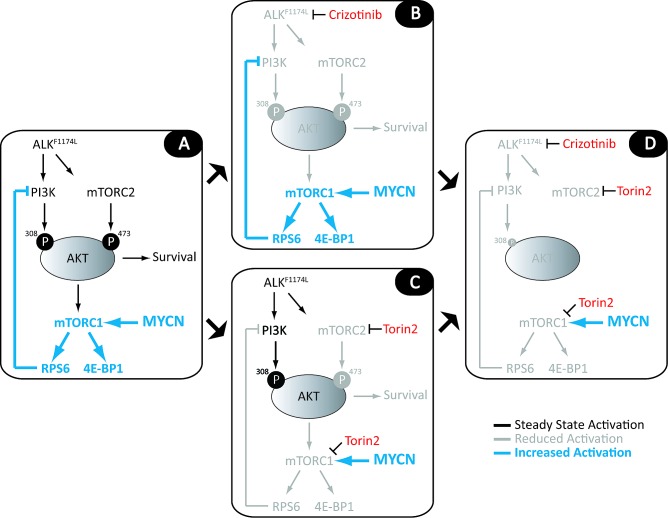
Model for combined use of crizotinib and mTOR inhibitors in *ALK^F1174L^*-mutated NB cells with *MYCN* amplification A, In *ALK^F1174L^*-mutant NB cells, AKT is phosphorylated at T308 and S473 by ALK-activated PI3K and mTORC2, respectively, promoting cell survival, transcriptional activation through release of 4E-BP1 from eIF4, and ribosome biogenesis through activation of RPS6. Activation of RPS6 in turn leads to inhibition of PI3K, creating a feedback loop that permits fine-tuning of proliferative and survival signaling. In *MYCN*-amplified, *ALK^F1174L^*-mutated NB cells, MYCN drives cellular proliferation, leading to increased dependence on RPS6 and 4E-BP1 activity. B, Treatment of these cells with crizotinib as a single agent inadequately inhibits mutant ALK, leading to marginally reduced PI3K (pAKT^T308^) and mTORC2 (pAKT^S473^) activities and a modest reduction in cell survival. Although the reduction in pAKT levels leads to a minimal reduction of pRPS6 in *MYCN*-amplified cells, this response is not sustained, as deregulated MYCN maintains upregulated mTORC1 activity. C, Single-agent treatment with specific ATP-competitive mTOR inhibitors, such as Torin2, blocks both mTORC1 and mTORC2 activity, downregulating pRPS6 and pAKT^S473^, although amplified *MYCN* continues to promote persistent mTORC1 activation and some level of PI3K feedback inhibition. D, The combination of crizotinib and Torin2 in *MYCN*-amplified cells targets the activities of PI3K, mTORC1 and mTORC2 simultaneously; continued loss of feedback inhibition of PI3K activity via incomplete suppression of MYCN/mTORC1/RPS6 signaling synergizes with crizotinib-driven inhibition of ALK to downregulate PI3K activity and, in turn, AKT^T308^ phosphorylation.

Figure 6 shows the impact of the *ALK^F1174L^* mutation on PI3K/AKT/mTOR signaling in NB, and the opportunities these interactions afford for targeted therapy. Cells expressing mutant *ALK* rely on phosphorylation of AKT at T308 and S473 via ALK-activated PI3K and mTORC2, respectively, for growth and survival. Subsequent activation of mTORC1 and RPS6 leads to inhibition of PI3K, creating a feedback loop that is needed for fine-tuning of this survival mechanism (Fig. 6A). In this context, crizotinib by itself only marginally reduces the activities of PI3K and mTORC2 (Fig. 6B). In cells expressing both the *ALK^F1174L^* mutation and amplified *MYCN*, mTORC1 activity is maintained by deregulated MYCN (Fig. 6B). Here the addition of an mTOR inhibitor such as Torin2 (or any equivalent clinically available agent) results in a synergistic response in which the downregulation of mTORC1 complements the crizotinib-driven inhibition of ALK to suppress PI3K/AKT activity (Fig. 6D). In cells expressing mutant *ALK* without deregulated MYCN, the picture is less clear. Exclusive use of Torin2, although able to completely downregulate mTORC1 activity in the absence of MYCN, leads to a much more striking increase of AKT phosphorylation by PI3K. Such loss of feedback inhibition leading to reciprocal activation of upstream signaling is a major limitation of single-agent kinase inhibitor therapy and suggests that a better result would be obtained by combining crizotinib with an inhibitor that possesses potent activity against PI3K as well as mTOR.

Although downstream signaling in cancer cells depends largely on the specific cell type, mTORC1 activation is a prominent feature of a number of different cancers that develop resistance to upstream kinase inhibitors [24, 25]. In PIK3CA-mutant breast cancer cells, for example, high levels of residual pRPS6 after treatment with a specific PI3K inhibitor positively correlate with either intrinsic or acquired resistance to this agent, which can be reversed by addition of a specific mTORC1 inhibitor [24]. Similarly, mTORC1 activation predicts responsiveness to RAF or MEK inhibitors in BRAF-mutant melanoma; in tumors that do not respond, pRPS6 remains activated and combining mTORC1 inhibition with RAF or MEK inhibition induces a cytotoxic response [25]. These observations, together with our findings in NB cells, support aberrant activation of PI3K/mTOR as part of a mechanism of acquired and/or innate resistance to kinase inhibitors and raise the possibility of adding PI3K/mTOR inhibitors to upstream kinase inhibitors at the outset of therapy.

It is becoming increasingly clear that efforts to perturb the signaling networks in a tumor cell using a single upstream inhibitor are likely to be futile. Our data suggest a need for clinical trials of ALK inhibitors in combination with downstream pathway inhibitors to enhance the antitumor activity of the former compounds, in particular, their ability to overcome the resistance of *ALK^F1174L^*–mutated NB cells to crizotinib. The strategies described here could also benefit patients with *ALK*-translocated cancers, where the *F1174L* mutation modulates crizotinib resistance and the same signaling pathways that drive tumor cells without translocated *ALK* are active. Finally, such strategies may be useful in delaying or even forestalling the development of resistance to newer ALK inhibitors.

## MATERIALS AND METHODS

### Cell lines and inhibitors

Neuroblastoma cell lines were obtained from the ATCC, ECACC, or the Children's Oncology Group (COG). Kelly, LAN-1, NGP, SHEP, and SH-SY5Y human NB cells [2] were cultured in RPMI-1640 medium supplemented with 10% fetal bovine serum (FBS), 100 units/mL penicillin, and 100 μg/mL streptomycin. CHLA-20 human NB cells were cultured in IMDM media supplemented with 20% FBS, 100 units/mL penicillin, 100 μg/mL streptomycin, and 1X Insulin-Transferrin-Selenium (Gibco). Cell lines were routinely mycoplasma-tested and genotyped at the DFCI Core Facility. Crizotinib (PF-02341066) and PF-05212384 were obtained from Pfizer Inc. (New York, NY) through a materials transfer agreement. Torin1 and Torin2 were synthesized in Dr. Nathanael Gray's laboratory. Rapamycin, NVP-BEZ235, AZD8055, LY294002, and GDC-0941 were purchased from Selleckchem.com (Houston, TX).

### Gene expression and gene set enrichment analyses (GSEA)

RNA was extracted with TRIzol® reagent according to the manufacturer's protocol (Invitrogen) from Kelly NB cells treated in triplicate with either vehicle (DMSO) or crizotinib (4 μM) for 6 hours. Gene expression was evaluated using Affymetrix U133A DNA microarrays (Affymetrix Inc. Santa Clara, CA). Data analysis was performed with GenePattern software [26] and GSEA as described previously [27].

### Cell viability assay and drug combination analysis

Viability experiments were performed in triplicate in 96-well plates and repeated at least three times with the Cell Proliferation Kit I (Roche) or CellTiter-Glo Luminescent cell viability assay (Promega, Madison, WI), according to the manufacturer's instructions. The synergy/additivity analyses were performed in 384- or 96-well plate format in two to four replicates and viability measured using the CellTiter-Glo viability assay as per the manufacturer's protocol. Compounds were added simultaneously at the indicated fixed ratios, and synergy was assessed using Calcusyn software (Biosoft, Ferguson, MO), using the Chou-Talalay method [28]. The resulting concentration pairs at the indicated ratios were visualized using Prism (GraphPad Software) or by isobologram (Microsoft Xcel) [29].

### Immunoblotting

Cell lysis and immunoblotting were performed as previously described [2]. All proteins were detected by chemiluminescence. Antibodies for pALK^Y1604^ (3341), ALK (3333), pAKT^S473^ (9271), pAKT^T308^ (4056), AKT (9272), pRPS6^S240/244^ (2215), RPS6 (2217), MYCN (9405), GAPDH (2118), β-Actin (4967), β-Tubulin (2128), 4E-BP1 (9644), p4E-BP1^T37/46^ (9459), mTOR (2972), pmTOR^S2448^ (2971) and PARP (9542) were purchased from Cell Signaling Technologies. Densitometry was performed using the ImageJ software package and statistical significance calculated using GraphPad Prism.

### Plasmid transfections

The oligos for *ALK* shRNA #6 were synthesized as previously described [30], while those for shRNA #8 were designed using the program described in [31]. Oligos were cloned into the pLKO-1 lentiviral vector containing the ampicillin resistance cassette. *MYCN* shRNA constructs were purchased from the Broad Institute (#1-TRCN0000020694, #3-TRCN0000363425). The shRNA knockdown experiments *were* performed as previously described [2]. The *ALK^F1174L^* mutation was introduced into wild type *ALK* cDNA using the QuickChange II Site-Directed Mutagenesis Kit (Strata-gene), cloned into the MSCV vector backbone containing the puromycin selectable marker (Addgene), and transfected into 293T cells with helper plasmids for virus production. SHEP cells were transduced with *ALK^F1174L^*–positive retrovirus, followed by puromycin selection for at least 3 days. *MYCN* expression in SHEP cells was repressed with 1μg/ml doxycycline for 24 hr. For the *in vivo* studies, Kelly cells were transduced with virus containing the Luc-mCherry-puro plasmid as described above.

### Cell cycle and Annexin V analysis

Kelly cells were treated for 72 hrs. with synergistic/additive doses of crizotinib (200 nM), Torin2 (20 nM), or the combination and cell cycle analyses were performed as previously described [2] or Annexin V staining performed using the BD Pharmingen FITC Annexin V Apoptosis Detection Kit I according to manufacturer's protocol.

### Immunohistochemistry

Tumors from the subcutaneous xenograft model were harvested at sacrifice, fixed in 10% neutral buffered formalin and paraffin-embedded for histologic studies. Tissue sections were stained with hematoxylin and eosin for morphological analysis as previously described [6]. For immunohistochemistry, 5-μm sections were stained with antibodies to ALK (Ventana), pRPS6^S240/244^, and cleaved caspase 3 (Cell Signaling Technologies: 2215 and 9664, respectively) using standard methods, including heat-induced epitope retrieval with citrate buffer pH 6 for pRPS6^S240/244^ and cleaved caspase 3 or EDTA buffer for ALK.

### Xenograft studies

All animal experiments were performed following approval from the Institutional Animal Care and Use Committee of the DFCI. NSG (Nod *scid* gamma) mice were used for the *in vivo* tumor growth inhibition studies.

Subcutaneous model. Animals were injected with 5 x 10^6^ Kelly cells and monitored until tumors appeared. Tumor volumes were calculated using the spheroid formula. Mice were divided into four treatment groups with similar mean tumor volumes (~60 mm^3^, n=8 per group) and treated with vehicle, crizotinib (75 mg/kg) p. o. daily, Torin2 (20 mg/kg) p. o. daily, or a combination of the two compounds. Treatments were administered in 5 day “cycles”, each consisting of 5 days on and 5 days off, for a total of three cycles. Tumors were measured at least once weekly and mice were sacrificed when tumor volume reached 1500 mm^3^. Tumors were collected from three mice per group as they reached the volume threshold; half of the specimens were fixed in 10% neutral-buffered formalin, while the other half were snap frozen in liquid nitrogen.

Metastatic model. Fifty 6-week-old NSG mice were injected in the lateral tail vein with 5 x 10^6^ Kelly-Luc-mCherry-puro cells. Six days after injection, 3 randomly selected cages of mice were imaged to determine baseline tumor burden. Ten days after injection, all mice were imaged and divided into four treatment groups (8 animals/group) with similar mean bioluminescence intensities: vehicle (nuclease-free water, Ambion, AM9916), crizotinib at 100 mg/kg, Torin2 at 20 mg/kg, or the crizotinib/Torin2 combination. Single agent crizotinib and Torin2 were given orally once daily for 10 consecutive days. Mice receiving the combination were treated orally once daily for 6 days, with treatment then adjusted to 5-day on/off cycles, (days 1-6, 13-17, and 23-27) to avoid toxicity. Tumor burden and mouse weights were monitored every 3-5 days by bioluminescence as previously described [32].

## Supplementary Material Figures and Tables


