# Supplementary material for: Molecular rationale for the use of PI3K/AKT/mTOR pathway inhibitors in combination with crizotinib in ALK-mutated neuroblastoma
Source: Oncotarget. 2014 Aug 19;5(18):8737–49. doi: 10.18632/oncotarget.2372 (PMC4226718; doi:10.18632/oncotarget.2372)
Supplement: Supplementary file 1 [file oncotarget-05-8737-s001.pdf]

Molecular rationale for the use of PI3K/AKT/mTOR pathway inhibitors in combination with crizotinib in *ALK*-mutated neuroblastoma

Supplementary Material

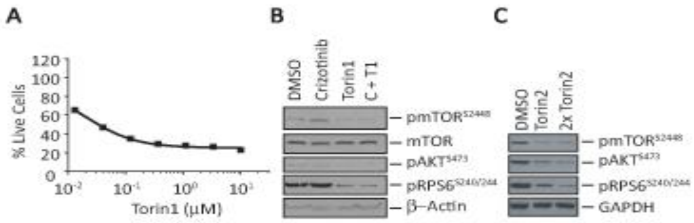

Figure S1: Addition of mTOR inhibitors leads to downregulation of mTOR activity in NB cells. A, Viability analysis of Kelly cells treated with increasing concentrations of Torin1. **B,** Western blot analysis of the indicated proteins in Kelly NB cells treated with crizotinib, Torin1 or the combination (C+T1) at the IC_{50} doses for 6 hr. **C,** Western blot analysis of the indicated phosphoproteins in Kelly cells treated with DMSO (control), Torin2 at IC_{50} doses or Torin2 at twice the IC_{50} doses for 6 hr.

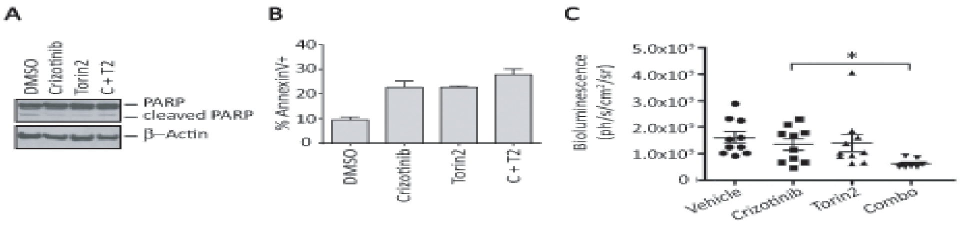

Figure S2: The combination of crizotinib and Torin2 in ALK-mutated, MYCN-amplified Kelly cells. **A**, Western blot analysis of Kelly cells treated with vehicle (DMSO), crizotinib, Torin2 or both (C+T2) for 72 hr. and immunoblotted with anti-PARP antibodies. Kelly cells treated with 2 mM crizotinib for 72 hr. served as a positive control. **B**, Quantification of Annexin V staining in Kelly cells following treatment with vehicle (DMSO), crizotinib, Torin2 or both (C+T2) for 72hr followed by flow cytometry analysis. **C**, Quantitation of tumor burden among the different treatment groups as measured by bioluminescence (crizotinib vs. combination, *P=0.026; ANOVA analysis).

Table S1: PI3K/AKT/mTOR gene sets enriched among differentially expressed genes following treatment of *ALK*^{F1174L}/*MYCN*-overexpressing NB cells with crizotinib

Gene Set	Size	NES	NOM p-val	FDR q-val
REACTOME AKT PHOSPHORYLATES TARGETS IN THE CYTOSOL	11	-1.4	0.047	0.467
REACTOME PI3K AKT ACTIVATION	26	-1.22	0.055	0.692
BIOCARTA MTOR PATHWAY	21	-1.22	0.197	0.685
REACTOME PIP3 ACTIVATES AKT SIGNALING	18	-1.17	0.224	0.753
REACTOME PI3K EVENTS IN ERBB4 SIGNALING	26	-1.09	0.273	0.807
REACTOME PI3K EVENTS IN ERBB2 SIGNALING	32	-1.04	0.491	0.832
PID IL2 PI3KPATHWAY	30	-1.01	0.451	0.843
BIOCARTA AKT PATHWAY	19	-1	0.432	0.85
PID PI3KCIAKTPATHWAY	28	-0.99	0.439	0.861
PID MTOR 4PATHWAY	57	-0.91	0.584	0.914
PARENT MTOR SIGNALING UP	491	-0.88	0.795	0.921
KEGG MTOR SIGNALING PATHWAY	55	-0.82	0.8	0.946
BIOCARTA IGF1MTOR PATHWAY	19	-0.69	0.917	0.982
XU HGF TARGETS INDUCED BY AKT1 6HR	17	-0.63	0.914	0.992

NES (normalized enrichment score); NOM p-val (nominal p-value); FDR q-val (false discovery rate ratio q-value)

Table S2: IC_{50} values of signaling pathway inhibitors in *ALK*^{F1174L}/*MYCN*-overexpressing Kelly NB cells

Pathway	Compound	IC_{50} (μM)
mTOR	Rapamycin	47.2
	Torin1	0.1
	AZD8055	0.11
	WYE-125132	0.13
	Torin2	0.02
PI3K	LY294002	3.0
	GDC-0941	0.4
PI3K/mTOR	NVP-BEZ235	0.36
	PF-04691502	0.16
	PF-05212384	0.01
